# Sodium thiosulfate attenuates glial-mediated neuroinflammation in degenerative neurological diseases

**DOI:** 10.1186/s12974-016-0488-8

**Published:** 2016-02-08

**Authors:** Moonhee Lee, Edith G. McGeer, Patrick L. McGeer

**Affiliations:** Kinsmen Laboratory of Neurological Research, University of British Columbia, 2255 Wesbrook Mall, Vancouver, BC V6T 1Z3 Canada

**Keywords:** NaSH, Neurotoxicity, TNFα, IL-6, Alzheimer’s disease, Parkinson’s disease

## Abstract

**Background:**

Sodium thiosulfate (STS) is an industrial chemical which has also been approved for the treatment of certain rare medical conditions. These include cyanide poisoning and calciphylaxis in hemodialysis patients with end-stage kidney disease. Here, we investigated the anti-inflammatory activity of STS in our glial-mediated neuroinflammatory model.

**Methods:**

Firstly, we measured glutathione (GSH) and hydrogen sulfide (H_2_S, SH^−^) levels in glial cells after treatment with sodium hydrosulfide (NaSH) or STS. We also measured released levels of tumor necrosis factor-α (TNFα) and interleukin-6 (IL-6) from them. We used two cell viability assays, MTT and lactate dehydrogenase (LDH) release assays, to investigate glial-mediated neurotoxicity and anti-inflammatory effects of NaSH or STS. We also employed Western blot to examine activation of intracellular inflammatory pathways.

**Results:**

We found that STS increases H_2_S and GSH expression in human microglia and astrocytes. When human microglia and astrocytes are activated by lipopolysaccharide (LPS)/interferon-γ (IFNγ) or IFNγ, they release materials that are toxic to differentiated SH-SY5Y cells. When the glial cells were treated with NaSH or STS, there was a significant enhancement of neuroprotection. The effect was concentration-dependent and incubation time-dependent. Such treatment reduced the release of TNFα and IL-6 and also attenuated activation of P38 MAPK and NFκB proteins. The compounds tested were not harmful when applied directly to all the cell types.

**Conclusions:**

Although NaSH was somewhat more powerful than STS in these in vitro assays, STS has already been approved as an orally available treatment. STS may therefore be a candidate for treating neurodegenerative disorders that have a prominent neuroinflammatory component.

**Electronic supplementary material:**

The online version of this article (doi:10.1186/s12974-016-0488-8) contains supplementary material, which is available to authorized users.

## Background

Sodium thiosulphate (Na_2_S_2_O_3_, STS) is an industrial compound which is typically available as the pentahydrate, Na_2_S_2_O_3_ · 5H_2_O. It also has medical uses in the treatment of some rare medical conditions. These include calciphylaxis in hemodialysis patients with end-stage kidney disease [1] as well as cyanide poisoning [2]. It also has functions as a preservative in table salt (less than 0.1 %) and alcoholic beverages (less than 0.0005 %). GMP products are widely available. While these amounts are very small, they indicate that the general population is consuming STS on a regular basis and increasing the dose may have important therapeutic applications.

Recently, STS was demonstrated to function as an anti-inflammatory agent [3]. For example, in acute liver failure induced in mice by lipopolysaccharide (LPS) or LPS/d-galactosamine, the survival rate was improved by hydrogen sulfide (H_2_S) and STS [4, 5]. STS is also reported to protect neuronal cells from ischemia [6].

This at least is partially due to the antioxidative function of these two agents; STS reacts with GSSG (oxidized glutathione) to produce reduced glutathione in the presence of hydroxyl radicals or peroxides. In addition, STS has a potential to produce hydrogen sulfide (H_2_S) by reaction with trans-sulfuration enzymes [7–10].

Previously, we reported that depletion of glutathione (GSH) in glial cells induces neuroinflammation resulting in neuronal death [11]. Neuroinflammation is, at least in part, characterized by the microglial release of proinflammatory factors such as cytokines and free radicals. Its purpose is to remove the source of harm so healing can take place. But when the inflammation is prolonged, it may cause neuronal dysfunction and death [12]. Chronic neuroinflammation is closely associated with the pathogenesis of several neurodegenerative diseases, including Alzheimer’s disease (AD) and Parkinson’s disease (PD).

In earlier studies, we demonstrated that H_2_S or H_2_S-releasing moieties, exhibited antioxidative, anti-inflammatory, and neuroprotective properties [13–15]. In this investigation, we studied the effects of sodium hydrosulfide (NaSH) or STS on SH-SY5Y neuronal cell death induced by supernatants from LPS and interferon-γ (IFNγ)-activated glial cells. These included cultured human astroglial and microglial cells and human THP-1 and U373 cells.

We found that NaSH and STS generated H_2_S and GSH in THP-1 and U373 cells, reduced release of proinflammatory cytokines such as tumor necrosis factor-α (TNFα) and interleukin-6 (IL-6) from LPS/IFNγ-stimulated microglia and THP-1 cells, as well as IFNγ-stimulated astrocytes and U373 cells. The toxicity was attenuated in a concentration-dependent and incubation time-dependent manner. This was due to reduced activation of intracellular inflammatory pathways such as P38 MAPK and NFκB proteins.

## Methods

### Materials

All reagents were purchased from Sigma (St. Louis, MO) unless stated otherwise. The following substances were applied to the cell cultures: bacterial LPS (*Escherichia coli* 055:B5) and human recombinant IFNγ (Bachem California, Torrance, CA). Sodium thiosulfate anhydrous (STS) was purchased from Sciencelab.com Inc. (Houston, TX).

### Cell culture and experimental protocols

The human monocyte THP-1 and astrocytoma U373 cell lines were obtained from the American Type Culture Collection (Manassas, VA). The human neuroblastoma SH-SY5Y cell line was a gift from Dr R. Ross, Fordham University, NY. These cells were grown in DMEM/F12 medium containing 10 % fetal bovine serum (FBS) and 100 IU/mL penicillin and 100 μg/mL streptomycin (Invitrogen, Carlsbad, CA) under humidified 5 % CO_2_ and 95 % air.

Human astroglial and microglial cells were isolated from surgically resected temporal lobe tissue as described previously [15]. Briefly, tissues were rinsed with a phosphate-buffered saline (PBS) solution and chopped into small (<2 mm^3^) pieces with a sterile scalpel. They were incubated in 10 mL of a 0.25 % trypsin solution at 37 °C for 20 min. Subsequently, DNase I (from bovine pancreas, Pharmacia Biotech, Baie d’Urfé, PQ, Canada) was added to reach a final concentration of 50 μg/mL. Tissues were incubated for an additional 10 min at 37 °C. After centrifugation at 275*g* for 10 min, the cell pellet was resuspended in the serum-containing medium and passed through a 100-μm nylon cell strainer (Becton Dickinson, Franklin Lakes, NJ). The cell suspension was then centrifuged (275*g* for 10 min), resuspended into 10 mL of Dulbecco’s modified Eagle medium (DMEM)-F12 with 10 % FBS containing gentamicin (50 μg/mL), and plated onto tissue culture plates (Becton Dickinson) in a humidified 5 % CO_2_, 95 % air atmosphere at 37 °C for 2 h. This achieved adherence of microglial cells. The non-adherent astrocytes along with myelin debris were transferred into new culture plates. Astrocytes adhered slowly and were allowed to grow by replacing the medium once a week. New passages of cells were generated by harvesting confluent astrocyte cultures using a trypsin–EDTA solution (0.25 % trypsin with EDTA, Invitrogen, Carlsbad, CA). Human astrocytes from up to the fifth passage from four surgical cases were used in the study.

**Fig. 1:**
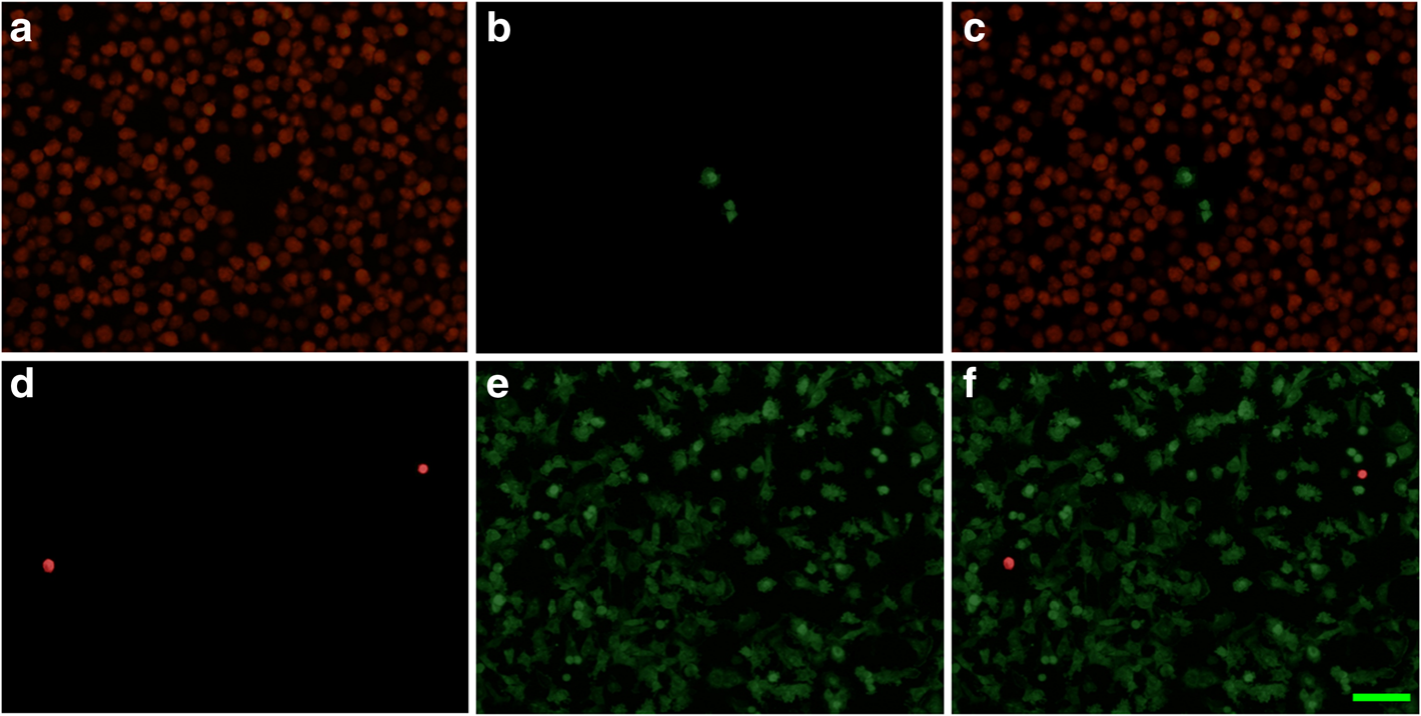
Immunofluorescence staining of Iba-1 (**a**, **d** *red*) in microglia and GFAP (**b**, **e** *green*) and merged images (**c**, **f**) in astrocytes in the microglial (**a**–**c**) and astrocytic cultures (**d**–**f**). The results suggested that more than 99 % of cells prepared from human brains are microglia and astrocytes, respectively. The calibration bar in F: 50 μm

For estimating the purity of astrocytic and microglial cell cultures, aliquots of the cultures were placed on glass slides at 37 °C for 48 h. The attached cells were then fixed with 4 % paraformaldehyde for 1 h at 4 °C and permeablized with 0.1 % Triton X-100 for 1 h at room temperature. After washing twice with PBS, the astrocytic culture slides were treated with a monoclonal anti-GFAP antibody (1/4,000, DAKO) and the microglial slides with the polyclonal anti-Iba-1 antibody (1/500, Wako Chemicals, Richmond, VA) for 3 h at room temperature. The slides were then incubated with Alexa Fluor 488-conjugated goat anti-mouse IgG antibody (Invitrogen, 1:500) and Alexa Fluor 546-conjugated goat anti-rabbit IgG antibody (Invitrogen, 1:500) in the dark for 3 h at room temperature to yield red fluorescence for Iba-1 positive cells and green fluorescence for GFAP positive cells. To visualize all cells, the slides were washed twice with PBS. Images were acquired using an Olympus BX51 microscope and a digital camera (Olympus DP71). Fluorescent images were colocalized with ImagePro software (Improvision Inc., Waltham, MA). We randomly chose 30 microscopic fields. Each field contained a total of about 500 cells. The numbers of astrocytes which appeared in microglial culture fields averaged 3.11 ± 0.12 cells per field. The numbers of microglia appearing in astrocytic culture fields was 2.05 ± 0.34 cells per field. The purity of microglia and astrocytic cultures was more than 99 % (Fig. 1).

To achieve SH-SY5Y differentiation, the undifferentiated cells were treated for 4 days with a high concentration of retinoic acid (RA, 5 μM) in DMEM/F12 medium containing 5 % FBS, 100 IU/mL penicillin, and 100 μg/mL streptomycin [16]. The RA-including medium was changed every 2 days. Differentiated SH-SY5Y cells demonstrated neurite extension, indicative of their differentiation [17], and act like a fully-differentiated human neuron-like cells [18].

### Experimental protocols

#### Protocol 1

Human astrocytes, U373 astrocytoma cells and THP-1 cells (5 × 10^5^ cells), and human microglial cells (5 × 10^4^ cells) were seeded into 24-well plates in 1 mL of DMEM/F12 medium containing 5 % FBS. NaSH or STS was then introduced at concentrations of 1 to 500 μM (the stock solutions were prepared just before use with sterilized deionized water). Incubation of the mixtures was carried out for 2, 4, 8, or 12 h. Cells were washed with PBS twice and replated in 800 μL DMEM/F12 medium containing 5 % FBS. One set of cells was then incubated at 37 °C for 2 days in the presence of inflammatory stimulants. For microglia and THP-1 cells, the stimulants were LPS at 1 μg/mL and IFNγ at 333 U/mL. For astrocytes and U373 cells, the stimulant was IFNγ alone at 150 U/mL. A comparable set of cells was incubated in media without inflammatory stimulants. After incubation, the supernatants (400 μL) were transferred to differentiated human neuroblastoma SH-SY5Y cells (2 × 10^5^ cells per well). The cells were incubated for a further 72 h and 3-(4,5-dimethylthiazol-2-yl)-2,5-diphenyl tetrazolium bromide (MTT) assays were performed as described below.

#### Protocol 2

Since it is perceived that both NaSH and STS could be useful as pharmaceutical agents, they must be shown to be nontoxic to humans. To determine whether they were directly affecting SH-SY5Y cell viability in the presence of LPS/IFNγ-stimulated THP-1 conditioned medium (CM) or IFNγ-stimulated U373 CM, NaSH or STS was added to a glial cell supernatant (400 μL) just before the supernatants were added to the SH-SY5Y cells. The glial cell supernatants were from THP-1 cells or U373 cells that had been activated for 2 days with the inflammatory stimulants previously described. The subsequent procedures were the same as in protocol 1.

### SH-SY5Y cell viability assays

The viability of SH-SY5Y cells following incubation with glial cell supernatants was evaluated by MTT assays as previously described [19]. Briefly, the viability was determined by adding MTT to the SH-SY5Y cell cultures to reach a final concentration of 1 mg/mL. Following a 1 h incubation at 37 °C, the dark crystals formed were dissolved by adding a SDS/DMF extraction buffer (300 μL, 20 % sodium dodecyl sulfate, 50 % *N*,*N*-dimethylformamide, pH 4.7). Subsequently, plates were incubated overnight at 37 °C, and optical densities at 570 nm were measured by transferring 100 μL aliquots to 96-well plates and using a plate reader with a corresponding filter. Data are presented as a percentage of the values obtained from cells incubated in fresh medium only.

The viability of differentiated SH-SY5Y cells was investigated by the lactate dehydrogenase (LDH) release assay [19]. Briefly, cell culture supernatants (100 μl) were pipetted into the wells of 96-well plates, followed by the addition of 15 μl lactate solution (36 mg/ml in PBS) and 15 μl p-iodonitrotetrazolium violet (INT) solution (2 mg/ml in PBS). The enzymatic reaction was started by addition of 15 μl of NAD^+^/diaphorase solution (3 mg/ml NAD^+^; 2.3 mg solid/ml diaphorase). After 1 h, optical densities were measured with a Model 450 microplate reader (Bio-Rad Laboratories, Richmond, CA) using a 490-nm filter. The amount of LDH released was expressed as a percentage of the value obtained in comparative wells where cells were 100 % lysed by 1 % Triton X-100.

### Measurement of TNFα and IL-6 release

Cytokine levels were measured in cell-free supernatants following 48 h incubation of THP-1 cells, U373 cells, microglial cells, and astrocytes. The cell stimulation protocols in these experiments were the same as that used in protocol 1. Quantitation was performed with ELISA detection kits (Peprotech, NJ) following protocols described by the manufacturer.

For determining TNFα and IL-6 depletion, protocols published in earlier studies were followed [17, 20]. Briefly, microglia were exposed to LPS/IFNγ, and astrocytes to IFNγ, for 2 days. Their supernatants were transferred to 24-well plates which had been coated with anti-TNFα or anti-IL-6 antibodies (10 mg/ml). After 3-h incubation, the supernatants were transferred to SH-SY5Y cells. After incubation at 37 °C for 3 days, MTT assays were performed on the SH-SY5Y cells.

### Activation of P38 MAPK and NFκB protein by Western Blotting

Western blotting on cell lysates was performed as described previously [19]. Briefly, microglia and astrocytes were exposed to NaSH and STS at 100 μM for 8 h and were subsequently exposed to stimulants for 2 h. Human microglia and astrocytes were treated with a lysis buffer (150 mm NaCl, 12 mm deoxycholic acid, 0.1 % Nonidet P-40, 0.1 % Triton X-100, and 5 mm Tris-EDTA, pH 7.4). The protein concentration of the cell lysates was then determined using a BCA protein assay reagent kit (Pierce, Rockford, IL). Proteins in each sample were loaded onto gels and separated by 10 % sodium dodecyl sulfate polyacrylamide gel electrophoresis (SDS-PAGE) (150 V, 1.5 h). The loading quantities of lysate proteins were 100 μg. Following SDS-PAGE, proteins were transferred to a PVDF membrane (Bio-Rad, CA) at 30 mA for 2 h. The membranes were blocked with 5 % milk in PBS-T (80 mm Na_2_HPO_4_, 20 mm NaH_2_PO_4_, 100 mm NaCl, 0.1 % Tween 20, pH 7.4) for 1 h and incubated overnight at 4 °C with a polyclonal anti-phospho-P38 MAP kinase antibody (9211, Cell Signaling, Beverly, MA, 1/2,000) or anti-phospho-P65 NFκB antibody (3031, Cell Signaling, 1/1,000). The membranes were then treated with a horseradish peroxidase-conjugated anti-IgG (P0448, DAKO, Mississauga, Ontario, CA, 1:2,000) or the secondary antibody anti-mouse IgG (A3682, Sigma, 1/3,000) for 3 h at room temperature, and the bands were visualized with an enhanced chemiluminescence system and exposure to photographic film (Hyperfilm ECL™, Amersham Biosciences). Equalization of protein loading was assessed independently using α-tubulin as the housekeeping protein. The primary antibody was anti-α-tubulin (T6074, Sigma, 1/2,000) and the secondary antibody anti-mouse IgG (A3682, Sigma, 1/3,000). Primary antibody incubation was overnight at 4 °C, and the secondary antibody incubation was for 3 h at room temperature.

### Measurement of H_2_S Levels

H_2_S (HS^−^) levels were measured using a previously described method [15]. To suppress endogenous production of H_2_S by CBS, all experiments were done with 0.1 mM of the specific CBS inhibitor hydroxylamine added to the solutions. Two sets of experiments were conducted: one in which the THP-1 cells and U373 cells were unstimulated and a second where they were stimulated with inflammatory mediators for 48 h. For THP-1 cells, the stimulation was LPS at 1 μg/ml and IFNγ at 333 U/ml, and for U373 cells, it was IFNγ at 150 U/ml. The cells in each case were treated with hydroxylamine plus NaSH or hydroxylamine plus STS (100 μM each) for 2, 4, 8, and 12 h. Following treatment, they were homogenized in 250 μl of ice-cold 100 mM potassium phosphate buffer (pH 7.4) containing trichloroacetic acid (10 % *w*/*v*). Zinc acetate (1 % *w*/*v*, 250 μl) was injected to trap the generated H_2_S. A solution of *N*,*N*-dimethyl-*p*-phenylenediamine sulfate (20 μM; 133 μl) in 7.2 m HCl and FeCl_3_ (30 μM; 133 μl) in 1.2 M HCl was added. Absorbance at 670 nm of the resulting mixture (300 μl) was determined after 10 min using a 96-well microplate reader (Bio-Rad). The H_2_S concentration of each sample was calculated against a calibration curve of NaSH (1–500 μmol/ml). The protein concentration was measured with a BCA protein assay reagent kit (Pierce, Rockford, IL). The concentrations were expressed as micromole per gram protein.

### Glutathione level

The GSH level was assessed by the method of Hissin and Hilf [21] and Lee et al. [11]. This assay detects reduced GSH by its reaction with *o*-phthalaldehyde (OPT) at pH 8.0. Cells (10^6^) in 1.5-ml tubes were washed twice with PBS, and 200 μl of 6.5 % trichloroacetic acid (TCA) was added. The mixture was incubated on ice for 10 min and centrifuged (13,000 rpm, 1 min). The supernatant was discarded, and the pellets were resuspended in 200 μl of ice-cold 6.5 % TCA and centrifuged again (13,000 rpm, 2 min). Supernatants (7.5 μl) were transferred to 96-well plates containing 277.5 μl phosphate-EDTA buffer (pH 8.0) in 1 M NaOH solution. Then, 15 μl OPT (1 mg/ml in methanol) was added. The reaction mixture was incubated in the dark at room temperature for 25 min. The fluorescence at 350 nm excitation/420 nm emission was measured in a multiwell plate reader. The concentration was calculated from a standard curve using serial dilutions of reduced GSH. The concentration was expressed as micromole per gram protein.

### Data analysis

The significance of differences between data sets was analyzed by one-way or two-way ANOVA tests. Multiple group comparisons were followed by a post hoc Bonferroni test. *P* values are given in the figure legends.

## Results

**Fig. 2:**
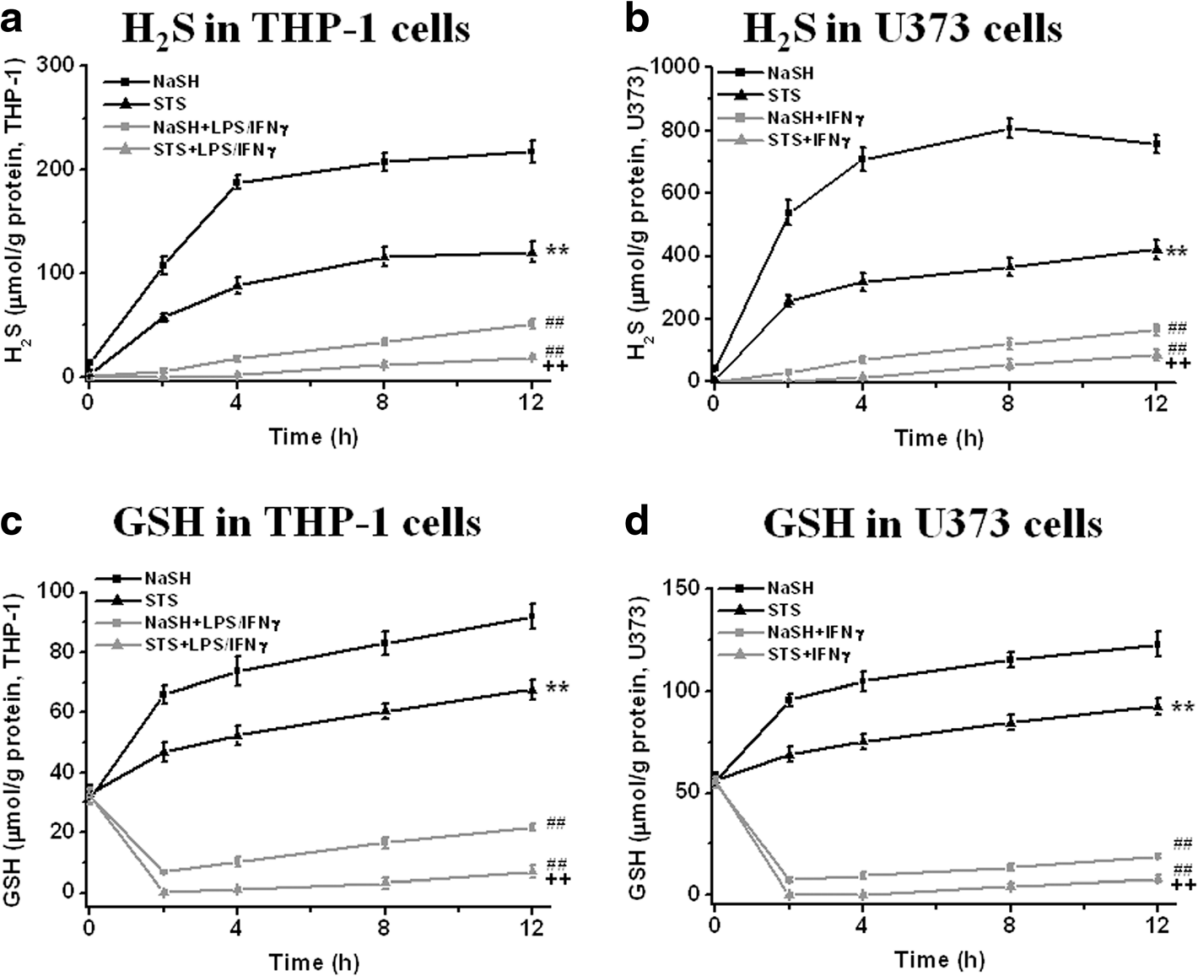
Intracellular H_2_S (SH^−^) and GSH concentrations produced from NaSH and STS in THP-1 cells (**a**, **c**) and U373 cells (**b**, **d**) with or without stimulation for 2 days (for THP-1 cells: LPS/IFNγ and for U373 cells: IFNγ). NaSH and STS (100 μM each) were separately added to THP-1 cells. See “Methods” section for details. Values are mean ± SEM, *n* = 4. Two-way ANOVA was carried out to test significance. Multiple comparisons were followed with post hoc Bonferroni tests. **a**–**d** **P < 0.01 for STS-treated groups compared with NaSH groups in the same condition (without stimulants) between 2 and 12 h, ^##^ *P* < 0.01 for LPS/IFNγ- or IFNγ-activated groups compared with groups without stimulants between 2 and 12 h, ^++^ *P* < 0.01 for STS-treated groups compared with NaSH groups in the presence of stimulants between 2 and 12 h. Activation of both cells with stimulants dramatically reduced H_2_S and GSH. NaSH was a somewhat more powerful agent than STS, but there was no qualitative difference between the two agents

In these experiments, we compared NaSH with sodium thiosulfate (STS). We firstly measured H_2_S (SH^−^) and GSH levels in THP-1 and U373 cells after treatment with STS or NaSH. These cells are regarded as surrogate cells of microglia and astrocytes. Intracellular H_2_S (SH^−^) and GSH concentrations were measured after treatment with LPS/IFNγ for THP-1 cells and IFNγ for U373 cells (100 μM each). STS and NaSH were exposed to the cells for 0, 2, 4, 8, and 12 h. The results are shown in Fig. 2. In both cell types, the H_2_S (SH^−^) and GSH content increased in an incubation time-dependent manner. However, the levels were dramatically decreased when THP-1 cells were exposed to LPS/IFNγ, and U373 cells to IFNγ, for 2 days. For the NaSH groups, LPS/IFNγ treatment reduced both H_2_S and GSH content in THP-1 cells by 75 % after 12 h. IFNγ treatment reduced H_2_S and GSH by 80 and 85 %, respectively, in U373 cells after 12 h. For STS groups, LPS/IFNγ treatment reduced H_2_S and GSH by 75 and 90 %, respectively, in THP-1 cells after in 12 h, and IFNγ treatment reduced by 80 % in H_2_S and by 90 % in GSH in U373 cells after 12 h. NaSH generated somewhat more H_2_S (SH^−^) and GSH than STS in both unstimulated and stimulated cells. The data indicate that 12 h after NaSH or STS treatment, substantial amounts of H_2_S (SH^−^) and GSH were still found in both types of cells after 2 days. Therefore, we chose 2, 4, 8, and 12 h incubation time periods for further experiments.

### Release of inflammatory cytokines

**Fig. 3:**
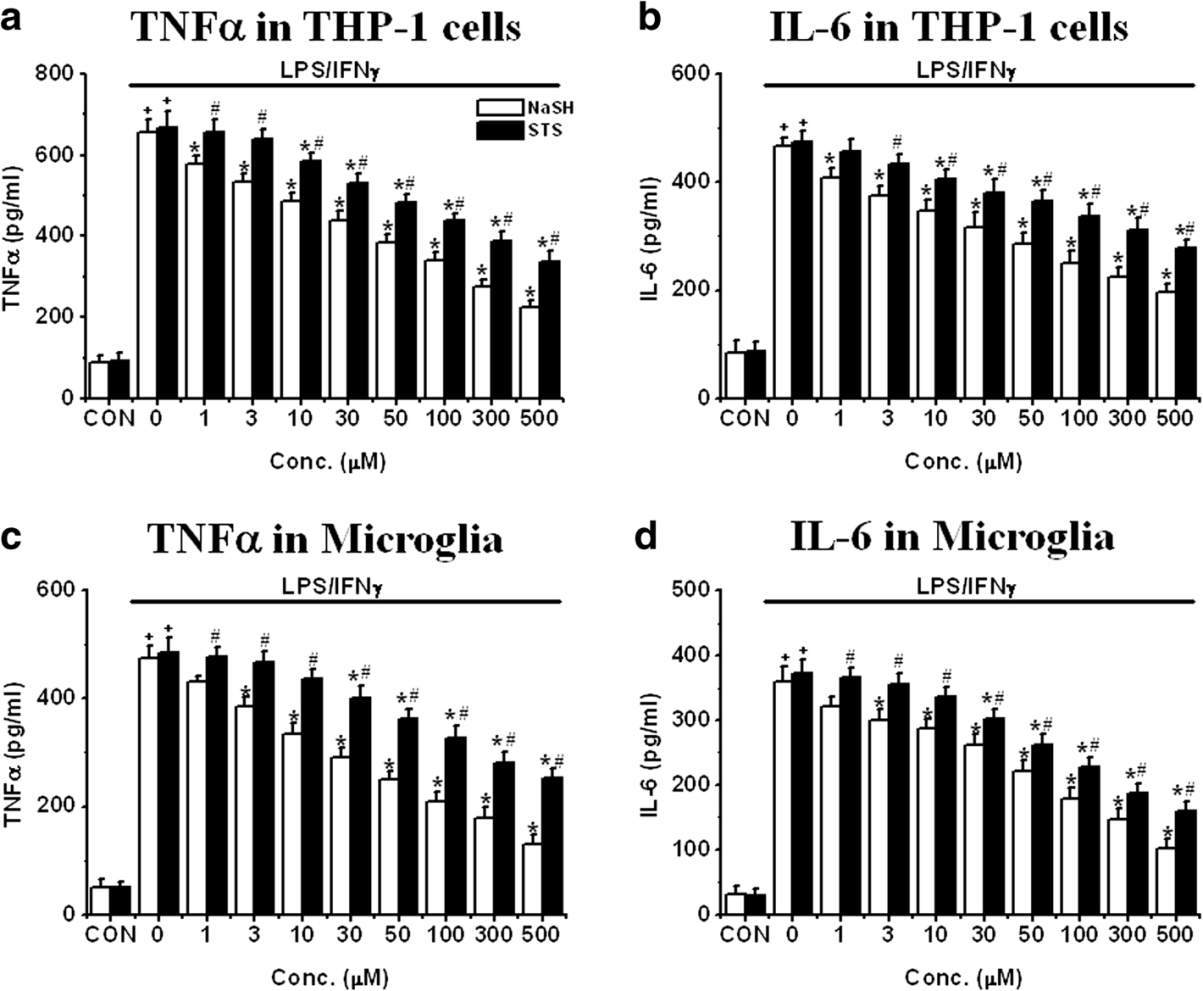
Effect of pre-treatment with NaSH or STS on released levels of TNFα (**a**, **c**) or IL-6 (**b**, **d**) from LPS/IFNγ-activated THP-1 cells (**a**, **b**) or LPS/IFNγ-activated microglia (**c**, **d**). 8 h pre-incubation with NaSH or STS was performed before LPS/IFNγ was exposed to the cells for 2 days. Values are mean ± SEM, *n* = 4. **a**, **b** Two-way ANOVA was carried out to test significance. Multiple comparisons were followed with post hoc Bonferroni tests. ^+^ *P* < 0.01 for LPS/IFNγ-activated groups compared with control (CON) groups in each condition, *P < 0.01 for NaSH- or STS-treated groups compared with LPS/IFNγ-activated groups, ^#^ *P* < 0.01 for STS-treated groups compared with NaSH groups in each condition. Note that both compounds attenuated released levels of TNFα and IL-6 from both cell types in a concentration-dependent manner

Inflammatory stimulation of microglia or THP-1 cells causes them to release the inflammatory cytokines TNFα and IL-6 [22, 23]. Figure 3 shows the effect on TNFα release by treatment of glial cells with NaSH and STS (1–500 μM for 8 h pre-incubation, protocol 1). THP-1 release of TNFα (Fig. 3a) and IL-6 (Fig. 3b) and human microglial release of TNFα (Fig. 3c) and IL-6 (Fig. 3d) are illustrated. The release of TNFα (Fig. 3a) and IL-6 (Fig. 3b) was reduced by NaSH and STS exposure in a concentration-dependent manner (Fig. 3, NaSH: *P* < 0.01 for 1 μM or higher and for STS: *P* < 0.01 for 10 μM or higher). The inhibitory effects of NaSH were more powerful than STS (*P* < 0.01 for 1 μM or higher and for TNFα and *P* < 0.01 for 3 μM or higher for IL-6). The IC_50_ values for NaSH and STS shown in Table 1 (A, B) were confirmatory. The pattern was similar in microglia (Fig. 3c, d). LPS/IFNγ stimulation caused a 9.5-fold increase of TNFα and an 11-fold increase of IL-6. Treatment with NaSH and STS reduced this release (NaSH: 75 % and STS: 50–60 % at 500 μM, *P* < 0.01).

**Table 1:** IC_50_ (μM) based on stimulated glial cell-released TNFα and IL-6 data of NaSH and STS at 8 h pre-incubation times

(A) TNFα: THP-1 cells and microglia				
	THP-1 cells		Microglia	
Pre-incubation time	NaSH	STS	NaSH	STS
8 h	31.15 ± 3.66	424.53 ± 18.25*	26.71 ± 4.32	579.53 ± 27.75*
(B) IL-6: THP-1 cells and microglia				
	THP-1 cells		Microglia	
Pre-incubation time	NaSH	STS	NaSH	STS
8 h	63.84 ± 7.95	621.53 ± 24.25*	33.36 ± 5.32	87.28 ± 6.79*
(C) IL-6: U373 cells and astrocytes				
	U373 cells		Astrocytes	
Pre-incubation time	NaSH	STS	NaSH	STS
8 h	432.26 ± 54.14	1356.53 ± 224.25*	7.44 ± 0.87	102.79 ± 11.25*

**Fig. 4:**
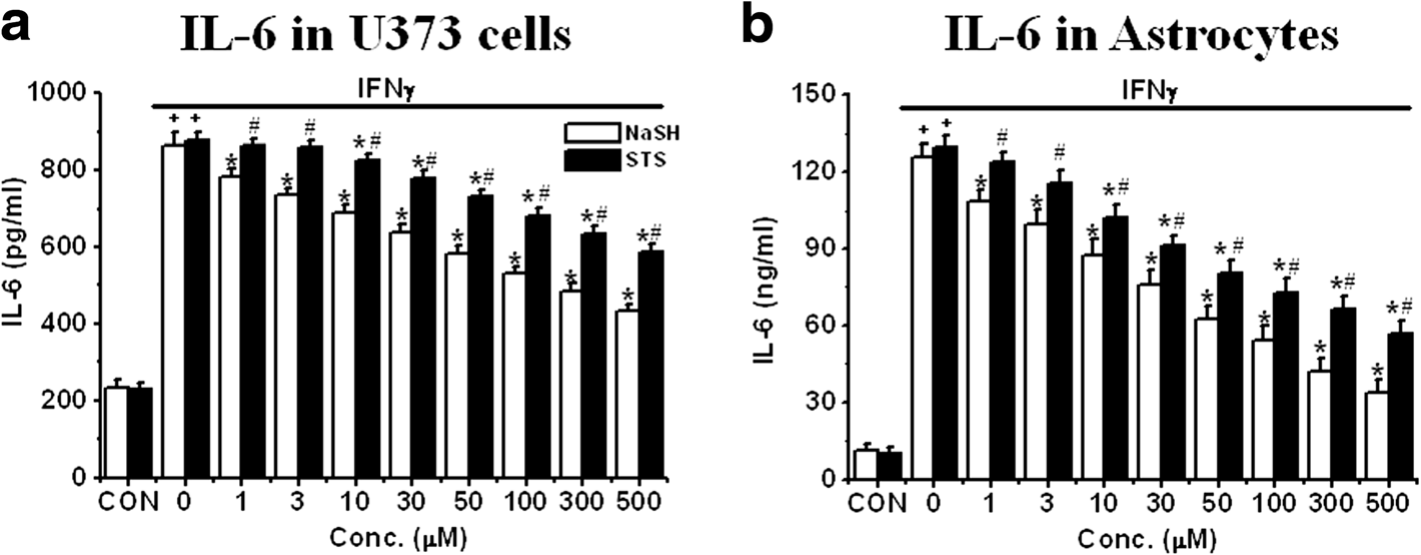
Effect of pre-treatment with NaSH or STS on released levels of IL-6 from IFNγ-activated U373 cells (**a**) or IFNγ-activated astrocytes (**b**). 8 h pre-incubation with NaSH or STS was performed before IFNγ was exposed to the cells for 2 days. Values are mean ± SEM, *n* = 4. **a**, **b** Two-way ANOVA was carried out to test significance. Multiple comparisons were followed with post hoc Bonferroni tests. ^+^ *P* < 0.01 for LPS/IFNγ-activated groups compared with CON groups for each condition, *P < 0.01 for NaSH- or STS-treated groups compared with LPS/IFNγ-activated groups, ^#^ *P* < 0.01 for STS-treated groups compared with NaSH groups in each conditions. Note that both compounds attenuated released levels of IL-6 from both cell types in a concentration-dependent manner

The tables summarized the IC_50_ results of the studies in Fig. 3 (Table 1 (A, B)) and Fig. 4 (Table 1 (C)). Values are mean ± SEM, *n* = 4. One-way ANOVA was carried out to test significance. Note that there was a significant reduction in IC_50_ values of NaSH compared with those of STS in all groups

*P < 0.01

For astrocytes, IL-6 is the main inflammatory mediator that is generated [24]. Figure 4 shows comparable data for IL-6 release from U373 cells (Fig. 4a) and cultured astrocytes (Fig. 4b). Cells were activated with IFNγ according to experimental protocol 1 and were then treated similarly to the THP-1 and microglial cells shown in Fig. 3. The release of IL-6 was more than fourfold higher in primary cultured astrocytes compared with U373 cells. However, both compounds reduced the release of IL-6 from IFNγ-activated U373 cells or astrocytes in a concentration-dependent manner (*P* < 0.01 NaSH: *P* < 0.01 from 1 μM and for STS: *P* < 0.01 from 10 μM). Again, NaSH is stronger in reducing IL-6 release in both cell types (*P* < 0.01 from 1 μM). The IC_50_ values for NaSH and STS are shown in Table 1 (C). NaSH is more potent than STS in attenuating release of these cytokines.

**Fig. 5:**
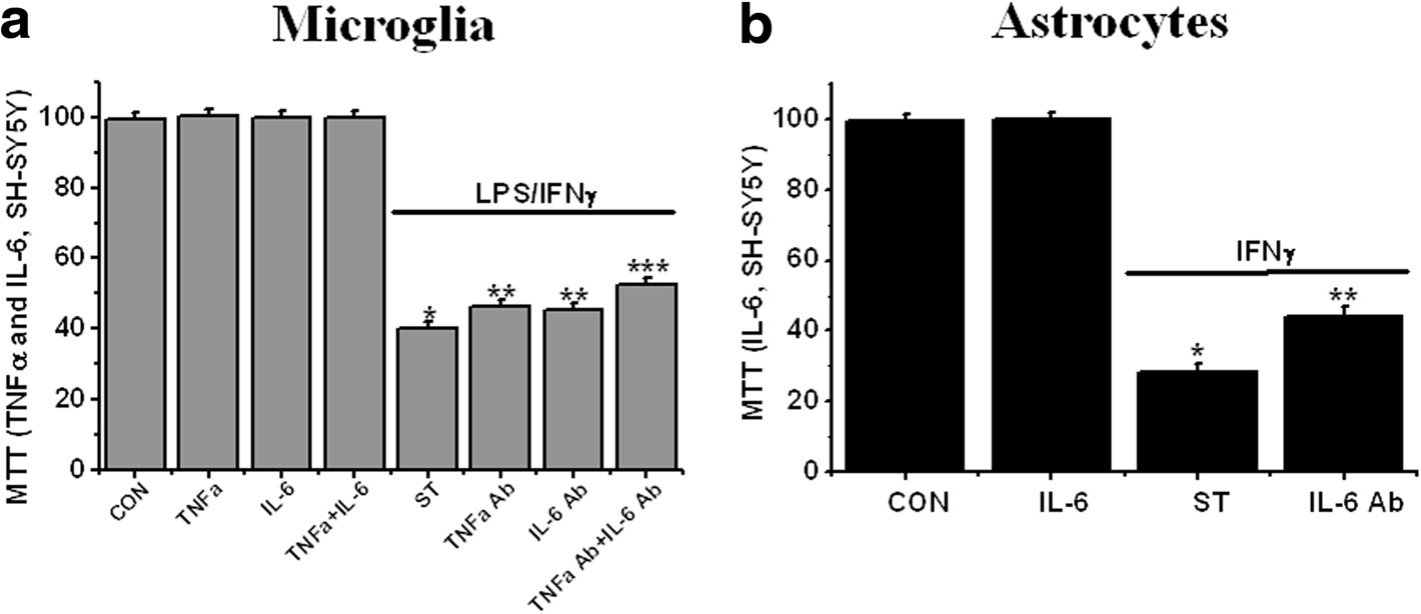
**a** Contribution of TNFα and IL-6 released from LPS/IFNγ-stimulated microglia on SH-5Y5Y cell viability. Direct treatment of RA-differentiated SH-SY5Y cells with TNFα (800 pg/ml) and/or IL-6 (600 pg/ml) was not changed SH-SY5Y cell viability (TNFa, IL-6, and TNFa + IL-6 in *x*-axis). Effects of removing TNFα and/or IL-6 using their specific antibody (ELISA methods) from neurotoxic secretions from microglia stimulated with LPS/IFNγ (TNFa Ab, IL-6 Ab, and TNFa Ab + IL-6 Ab in *x*-axis). CON and ST mean unstimulation and stimulation, respectively. See “Methods” section for details. One-way ANOVA was carried out to test significance. *P < 0.01 vs CON group, **P < 0.01 vs ST group, and ***P < 0.01 vs TNFa Ab and IL-6 Ab groups. **b** Contribution of IL-6 released from IFNγ-stimulated astrocytes on SH-5Y5Y cell viability. For details, see “Methods” section. Direct treatment of RA-differentiated SH-SY5Y cells with IL-6 (120 ng/ml) was not changed SH-SY5Y cell viability (IL-6 in *x*-axis). Effects of removing IL-6 using their specific antibody (ELISA methods) from neurotoxic secretions from astrocytes stimulated with IFNγ (IL-6 Ab in *x*-axis). CON and ST mean unstimulation and stimulation, respectively. See “Methods” section for details. One-way ANOVA was carried out to test significance. *P < 0.01 vs CON group and **P < 0.01 vs ST group

We investigated, as described in methods, the direct effects of TNFα and IL-6 treatment, alone and in combination, on the viability of SH-SY5Y cells. MTT assays were performed after 3 days incubation at 37 °C. There were no changes in viability as shown in Fig. 5. However, the effects were different when the cells were exposed to cytokine-depleted CM from microglia and astrocytes. For depleted microglial conditioned medium (CM), the SH-SY5Y viability was increased by 10.6 % for TNFα, 8.7 % for IL-6, and 21 % for TNFα + IL-6 (Fig. 5a). For astrocytes, the corresponding increase in viability for IL-6 depletion was 22 % (Fig. 5b).

### Neuroprotective effect of NaSH and STS against microglial, astrocytic, THP-1, and U373 cell toxicity

We investigated the effects of pre-treatment with NaSH and STS on the toxicity of CM toward SH-SY5Y cells. The CM was obtained by 2 days treatment of LPS/IFNγ-activated THP-1 cells and IFNγ-activated U373 cells. Experimental protocol 1 was followed.

**Fig. 6:**
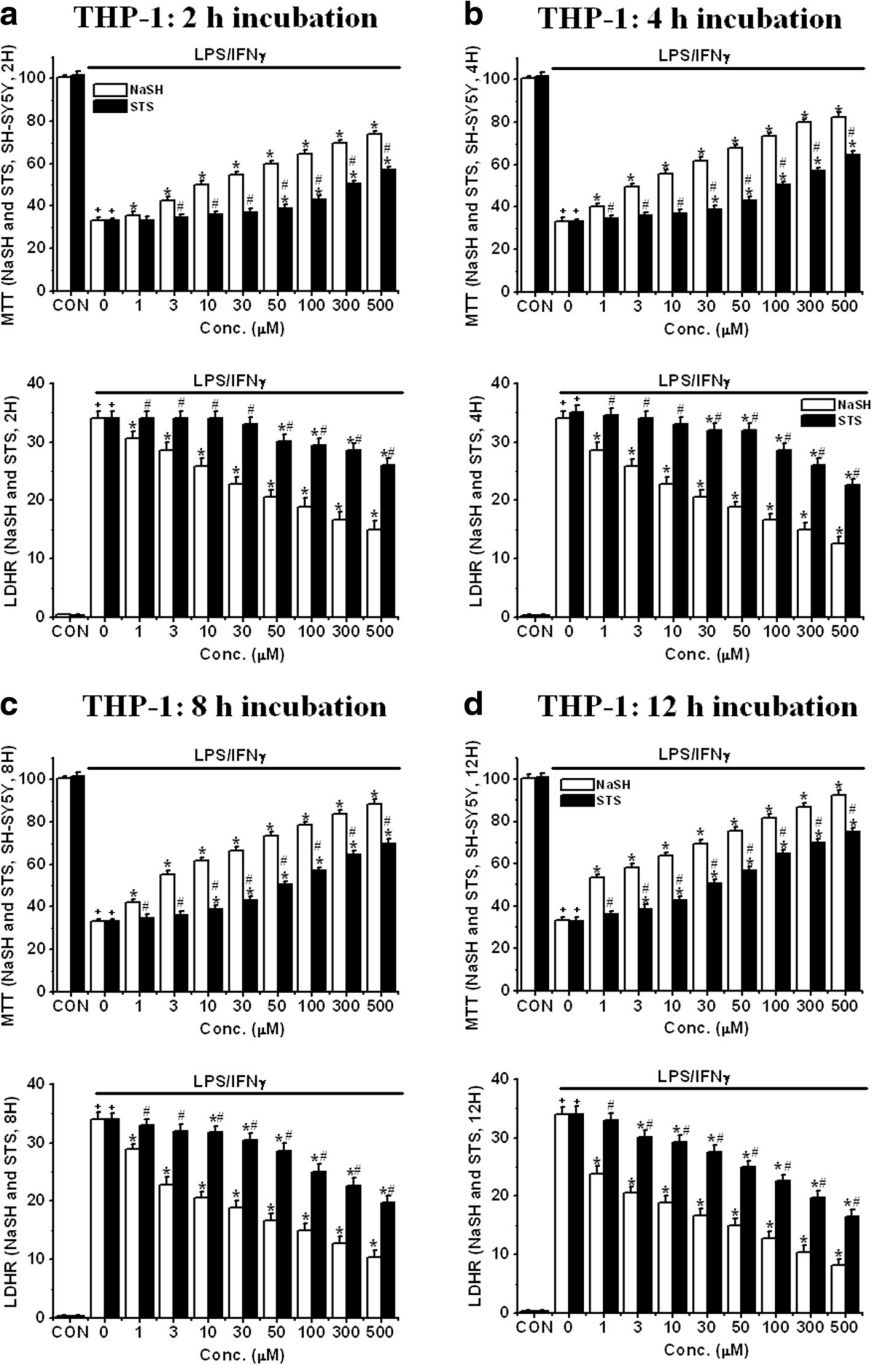
Effect of treatment with NaSH or STS on SH-SY5Y cell viability changes induced by LPS/IFNγ-activated THP-1 cell conditioned medium (CM) as followed by MTT and lactate LDH release assays (protocol 1). **a** 2 h pre-incubation, **b** 4 h pre-incubation, **c** 8 h pre-incubation, and **d** 12 h pre-incubation. *Upper panel*: MTT assay data and *lower panel*: LDH release assay data. Values are mean ± SEM, *n* = 4. Two-way ANOVA was carried out to test significance. Multiple comparisons were followed with post hoc Bonferroni tests. ^**+**^ *P* < 0.01 for LPS/IFNγ-activated groups compared with CON groups in the same concentrations, *P < 0.01 for NaSH- or STS-treated groups compared with LPS/IFNγ-activated groups, ^#^ *P* < 0.01 for STS-treated groups compared with NaSH roups in the same concentrations (**a**–**d**). Note that both compounds attenuated a reduction of SH-SY5Y cell viability induced by LPS/IFNγ-activated THP-1 cell CM in a concentration-dependent and pre-incubation time-dependent manner

It was found that NaSH and STS each attenuated SH-SY5Y cell viability loss by THP-1 CM (Fig. 6). The effect was concentration-dependent in the 1–500 μM range and was time-dependent up to 12 h (Fig. 6a: 2 h pre-incubation, 6b: 4 h pre-incubation, 6c: 8 h pre-incubation and 6d: 12 h pre-incubation). At the shortest time interval of 2 h and at the lowest concentration of 1 μM, the protective effect of NaSH was minimal. But the toxicity was reduced to about half at a concentration of 500 μM (*P* < 0.01 from 1 μM). By 12 h the protective effect had increased to the point where the lowest concentration reduced the toxicity by about one third while the highest concentration reduced it by about five- to sixfold. Pre-treatment with STS also attenuated THP-1 toxicity toward SH-SY5Y cells at 2 h pre-incubation (*P* < 0.01 from 50 μM) but was less effective than NaSH (*P* < 0.01 from 3 μM); protective levels were 75 % of those obtained with NaSH.

**Fig. 7:**
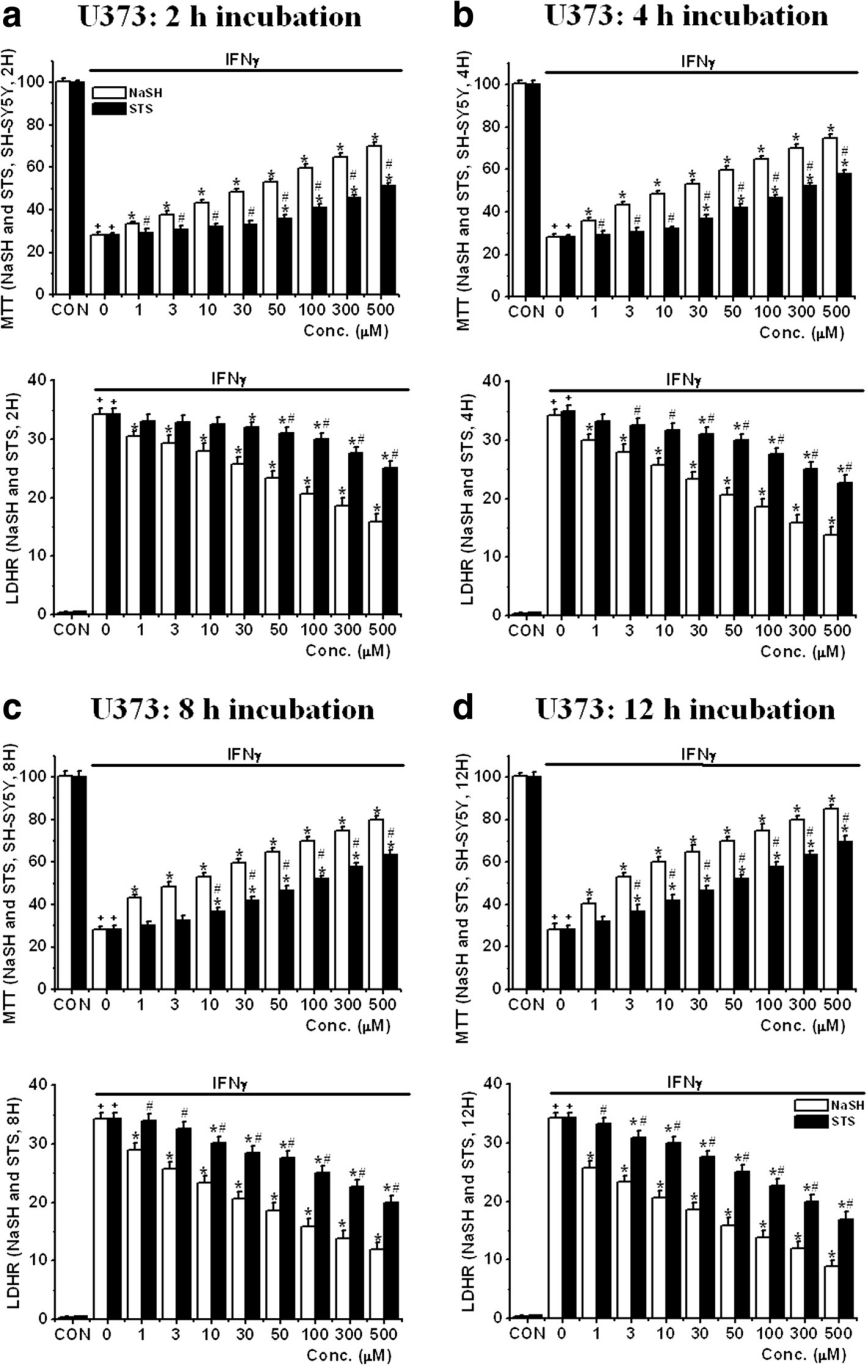
Effect of treatment with NaSH or STS on SH-SY5Y cell viability changes induced by IFNγ-activated U373 cell CM as followed by MTT and LDH release assays (protocol 1). **a** 2 h pre-incubation, **b** 4 h pre-incubation, **c** 8 h pre-incubation, and **d** 12 h pre-incubation. *Upper panel*: MTT assay data and *lower panel*: LDH release assay data. Values are mean ± SEM, *n* = 4. Two-way ANOVA was carried out to test significance. Multiple comparisons were followed with post hoc Bonferroni tests. ^+^ *P* < 0.01 for LPS/IFNγ-activated groups compared with CON groups in the same concentrations, *P < 0.01 for NaSH- or STS-treated groups compared with LPS/IFNγ-activated groups, ^#^ *P* < 0.01 for STS-treated groups compared with NaSH groups at the same concentrations (**a**–**d**). Note that both compounds attenuated the reduction of SH-SY5Y cell viability induced by IFNγ-activated U373 CM in a concentration-dependent and pre-incubation time-dependent manner

Figure 7 shows the effects of exposing SH-SY5Y cells to the neurotoxic CM from U373 cells that had been stimulated with 150 U of IFNγ according to experimental protocol 1. The figure shows highly similar data to those obtained from stimulated THP-1 cells (Fig. 7a: 2 h pre-incubation, 7b: 4 h pre-incubation, 7c: 8 h pre-incubation and 7d: 12 h pre-incubation) were obtained. Again, the protective effect of the both agents was both concentration-dependent and incubation time-dependent. NaSH was more neuroprotective than STS (MTT test data: *P* < 0.01 from 50 μM at 2 h, *P* < 0.01 from 30 μM at 4 h, *P* < 0.01 from 10 μM at 8 h, *P* < 0.01 from 3 μM at 12 h).

Table 2 summarized the IC_50_ results of these studies. As shown in Figs. 6 and 7, the concentrations of NaSH required to reach the level of IC_50_, for both THP-1 and U373 cells, are approximately threefold lower than those required for STS at all the incubation times in both THP-1 and U373 cells. The data indicate that IC_50_s of NaSH and STS are reduced with a longer pre-incubation time. The IC_50_s of NaSH were at least three times lower than those of STS in each pre-incubation time in both cell types.

**Table 2:** IC_50_ (μM) based on MTT (A-1 and B-1) and LDHR data (A-2 and B-2) of NaSH and STS at different pre-incubation times in THP-1 cells and U373 cells

(A) MTT data				
	THP-1 cells		U373 cells	
Pre-incubation time	NaSH	STS	NaSH	STS
2 h	28.55 ± 2.32	121.53 ± 3.25	28.55 ± 3.32	95.53 ± 3.25
4 h	23.83 ± 2.34	96.47 ± 2.44	22.43 ± 3.34	49.63 ± 2.44
8 h	10.35 ± 2.11	53.55 ± 2.46	9.44 ± 2.11	30.27 ± 2.46
12 h	5.83 ± 1.47	35.73 ± 1.78	4.47 ± 1.83	19.57 ± 1.78
(A) LDHR data				
	THP-1 cells		U373 cells	
Pre-incubation time	NaSH	STS	NaSH	STS
2 h	15.33 ± 0.88	173.53 ± 18.75	32.48 ± 2.32	98.38 ± 7.25
4 h	3.83 ± 0.56	100.47 ± 12.44	25.18 ± 2.21	71.05 ± 4.44
8 h	2.11 ± 0.16	61.81 ± 5.46	10.17 ± 1.11	47.91 ± 3.46
12 h	1.42 ± 0.22	33.51 ± 2.31	1.84 ± 0.28	32.22 ± 2.78

The tables summarized the IC_50_ results of the studies in Figs. 6 and 7. Values are mean ± SEM, *n* = 4. Two-way ANOVA was carried out to test significance. Multiple comparisons were followed with post hoc Bonferroni tests. Note that there was a significant reduction in IC_50_ values between any pre-incubation time groups and that there was a significant reduction in IC_50_ values of NaSH compared with those of STS in the same incubation time groups

**Fig. 8:**
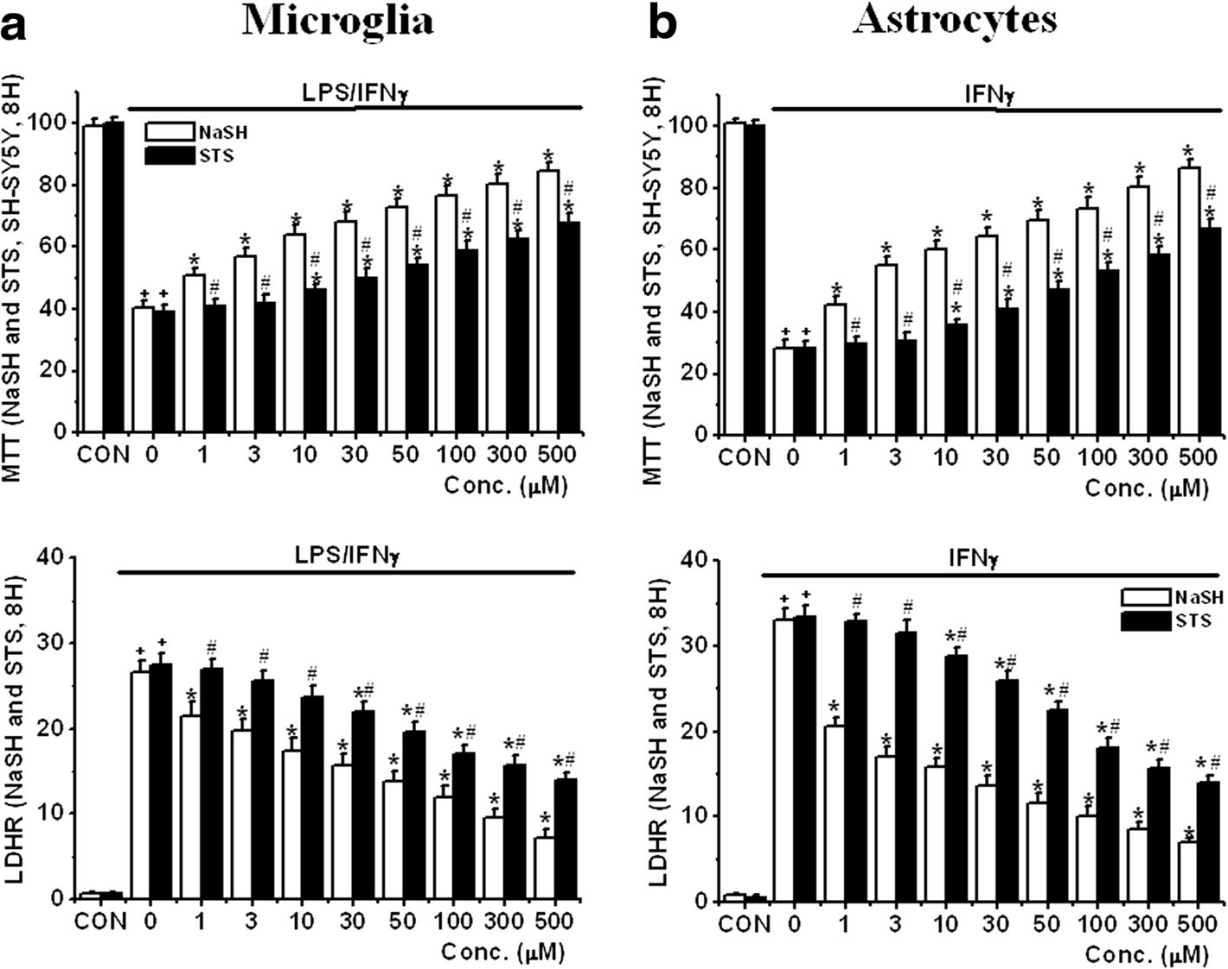
Effect of pre-treatment with NaSH or STS for 8 h on SH-SY5Y cell viability changes induced by **a** LPS/IFNγ-activated microglial CM and **b** IFNγ-activated astrocytic CM as followed by MTT and LDH release assays (protocol 1). *Upper panel*: MTT assay data and *lower panel*: LDH release assay data. Values are mean ± SEM, *n* = 4. Two-way ANOVA was carried out to test significance. Multiple comparisons were followed with post hoc Bonferroni tests. ^+^ *P* < 0.01 for LPS/IFNγ- (**a**) or IFNγ-activated (**b**) groups compared with CON groups in each condition, *P < 0.01 for NaSH- or STS-treated groups compared with LPS/IFNγ- or IFNγ-activated groups, ^#^ *P* < 0.01 for STS groups compared with NaSH groups in each condition. Note that both compounds attenuated a reduction of SH-SY5Y cell viability induced by LPS/IFNγ-activated THP-1 cell CM in a concentration-dependent manner

We also investigated the effect of supernatants from LPS/IFNγ-stimulated microglia and IFNγ-stimulated astrocytes on SH-SY5Y cell viability after treatment with all the agents. Due to the limited availability of microglia and astrocytes, they were pre-exposed to the compounds at only one time period (8 h) prior to treatment with stimulatory agents (experimental protocol 1). For measuring SH-SY5Y cell viability, the MTT and LDH release assays were utilized (upper panel: MTT assays and lower panel: LDH release assays). It was observed that both NaSH and STS reduced the toxicity of both LPS/IFNγ-stimulated microglia (Fig. 8a) and IFNγ-stimulated astrocytes (Fig. 8b) toward SH-SY5Y cells (MTT assay data: for NaSH: *P* < 0.01 from 1 μM and for STS: *P* < 0.01 from 10 μM). Again, NaSH had a greater neuroprotective effect than STS (MTT assay data: *P* < 0.01 from 1 μM) (Table 3). These data demonstrate that the effects observed with cultured microglia and astrocytes are comparable to those observed with the THP-1 and U373 cell lines.

**Table 3:** IC_50_ (μM) based on MTT (A-1 and B-1) and LDHR data (A-2 and B-2) of NaSH and STS at different pre-incubation times in microglia and astrocytes

Human microglia and astrocytes				
(B) MTT data				
	Miroglia		Astrocytes	
Pre-incubation time	NaSH	STS	NaSH	STS
8 h	7.62 ± 0.46	68.43 ± 3.25 *	9.01 ± 0.71	77.53 ± 3.48 *
(B) LDHR data				
	Miroglia		Astrocytes	
Pre-incubation time	NaSH	STS	NaSH	STS
8 h	23.12 ± 1.46	51.43 ± 3.25 *	1.32 ± 0.11	53.06 ± 4.27 *

The tables summarized the IC_50_ results of the studies shown in Fig. 8. Values are mean ± SEM, *n* = 4. One-way ANOVA was carried out to test significance. Note that there was a significant reduction in IC_50_ values of NaSH compared with those of STS

*P < 0.01

However, pre-treatment with NaSH or STS of THP-1 cells and U373 cells for 12 h and of microglia and astrocytes for 8 h with both compounds did not change the viability of any glial cells by stimulants (LPS/IFNγ for THP-1 cells and microglia: Additional file 1: Figure S1A and S1C, and IFNγ for U373 cells and strocytes: Additional file 1: Figure S1B and S1D).

It was also found that NaSH and STS were not directly protective of SH-SY5Y cells (Additional file 1: Figure S2). When they were added to the CM after stimulation had taken place (protocol 2, A: THP-1 cells and B: U373 cells), they had no effect. As the figure shows, there was no difference between the agents and there was no effect of concentration. This establishes that the agents were working by inhibiting the glial inflammatory response.

In summary, NaSH and STS were not toxic to the glial cells in the presence of inflammatory stimuli. The viability of SH-SY5Y cells treated with the CM from LPS/IFNγ-stimulated THP-1 and IFNγ-stimulated U373 cells was unchanged.

As further control experiments, we tested the effects of NaSH and STS on the growth and survival of human microglia and astrocytes under normal and inflammatory conditions. Human microglia and astrocytes were treated with NaSH or STS for 3 days. MTT assays were then performed on the cultures. Microglia and astrocytes were next treated with stimulants plus NaSH or STS for 2 days. The stimulants were LPS/IFNγ for microglia and IFNγ for astrocytes. Comparative MTT assays were then performed on the cultures. The results are shown in Additional file 1: Figure S3. It was found that STS and NaSH did not change the viability of microglia and astrocytes under any of the experimental conditions.

### Activation of intracellular inflammatory pathway in microglia and astrocytes

**Fig. 9:**
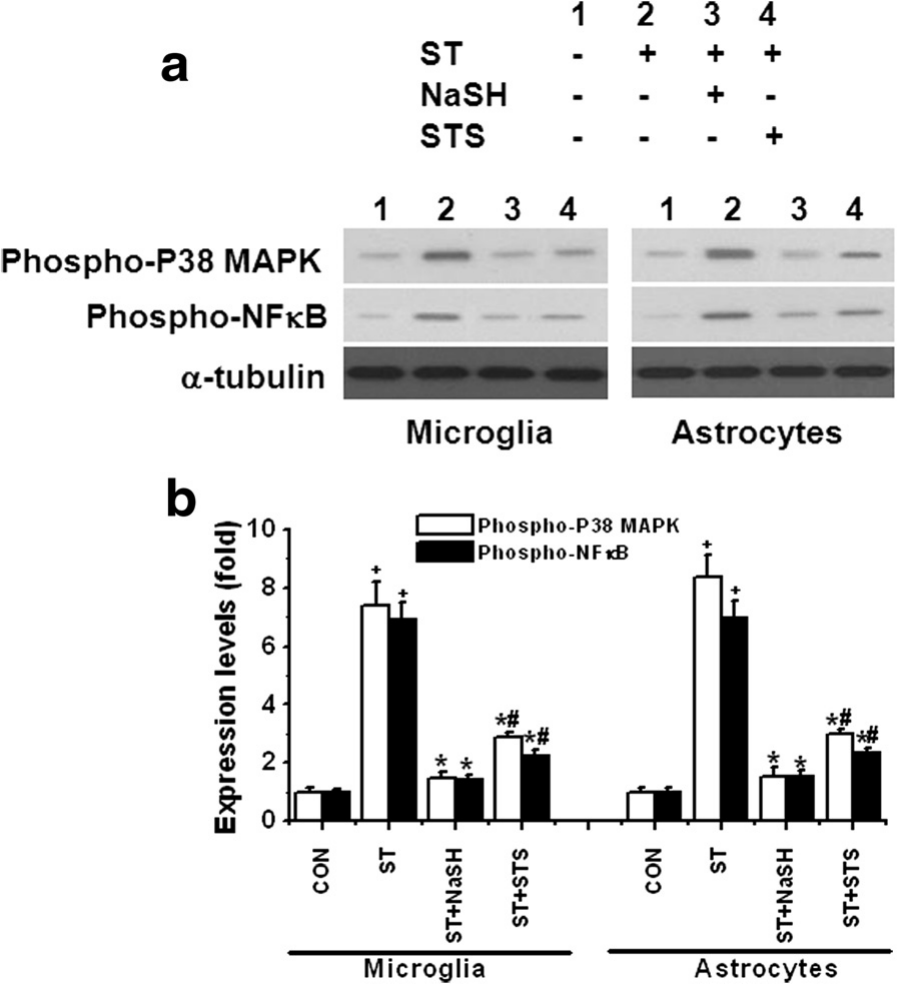
Effect of pre-treatment with NaSH or STS for 8 h on levels of phospho-P38 MAPK and phospho-P65-NFκB in LPS/IFNγ-activated human microglia **a** (*left panel*) and IFNγ-activated astrocytes **a** (*right panel*). Cell extracts were prepared and the proteins separated by SDS-PAGE. Representative blots are shown in **a** and quantitative results in **b**. To ensure equal loading, the densitometric value of each band was normalized to the corresponding band for α-tubulin. Values are mean ± SEM, *n* = 3. One-way ANOVA was carried out to examine the significance of differences. ^+^ *P* < 0.01 for LPS/IFNγ- or IFNγ-activated groups compared with CON groups in each condition, *P < 0.01 for NaSH- or STS-treated groups compared with LPS/IFNγ- or IFNγ-activated groups, ^#^ *P* < 0.01 for STS-treated groups compared with NaSH groups in each conditions. Note that both compounds attenuated the levels of phospho-P38 MAPK and phospho-P65-NFκB in both cells

In the final set of experiments, we investigated the effects of pre-treatment with NaSH and STS at 100 μM on phospho-P38 MAPK and phospho-NFκB proteins. These are indicative of intracellular inflammatory activation. The data are shown in Fig. 9. Upon exposure to the stimulants, both microglia and astrocytes showed an increase in these proteins. For microglia, P38 MAPK was increased sevenfold and NFκB eightfold. For astrocytes, P38 MAPK and NFκB were increased sevenfold. Both compounds attenuated these increases, and NaSH was again more powerful than STS (NaSH: 80 % reduction and STS: 60–65 % reduction).

## Discussion

Brain damage is known to cause neuroinflammation by activating microglia and astrocytes to release proinflammatory factors such as cytokines, toxic free radicals, and proteases. Many previous publications indicate that oxidative stress in neuronal cells plays an important role in their death [25]. Indeed, we first reported that depletion of GSH in both microglia and astrocytes induces neuroinflammation and results in neurotoxicity. It is known that H_2_S is a reducing agent which can increase intracellular glutathione levels. Glutathione is a major intracellular antioxidant [26] and thereby inhibits oxidative stress [11]. H_2_S activates adenylate cyclase to increase [cAMP]_i_ which has an inhibitory effect on transcription of inflammatory cytokine mRNAs [26]. All of these mechanisms may be contributing to the results reported here.

In this study, we have shown that STS has the same broad spectrum inhibition of toxic inflammatory activity as NaSH. Since neuroinflammation has been demonstrated to occur in degenerative neurological diseases such as Alzheimer’s disease (AD) and Parkinson’s disease (PD) [27, 28], STS is a potential therapeutic agent for these and other neurodegenerative disorders. It has already been approved as a treatment for arsenic poisoning and calciphylaxis in hemodialysis patients with end-stage kidney disease. This is at least partly due to an increase in H_2_S and GSH in microglia and astrocytes by STS [7–10].

In these studies, we observed that the longer pre-incubation time with STS and NaSH, the lower the concentrations of NaSH or STS to allow 50 % of SH-SY5Y cell survival (IC_50_s) when exposed to their CMs. This correlates with an increase in intracellular GSH and SH^−^ levels in both glial cell types (Fig. 2). Those increases inhibited the proinflammatory pathways involving P38 MAP kinase and NFκB proteins (Fig. 9). In turn, this led to production and release of the proinflammatory factors TNFα and IL-6 (Figs. 3 and 4). The overall result was a decrease in loss of SH-SY5Y cells in a concentration and pre-incubation time-dependent manner (Figs. 6, 7, and 8). We found that NaSH is more potent than STS (Tables 1, 2, and 3). This is apparently due to higher levels of intracellular GSH and SH^−^ reached by NaSH compared with STS treatment.

In our previous experiments, we found that TNFα and IL-6, alone and in combination, did not change SH-SY5Y cell viability (Fig. 5). However, they enhanced SH-SY5Y sensitivity to other inflammatory materials released from LPS/IFNγ-stimulated microglia and IFNγ-stimulated astrocytes [17, 20].

Murutani et al. [6], in HPLC-linked measurements, reported that treatment of SH-SY5Y cells with Na_2_S increased intracellular and extracellular levels of thiosulfate, but not H_2_S. In the current study, we measured H_2_S levels in THP-1 and U373 cells with the zinc acetate-mediated H_2_S-trap methods (i.e., methylene blue method). Given the known limitation of this method that utilizes highly acidic condition [29, 30], we were not able to measure thiosulfate levels in the glial cells in this study.

It is known that direct inhalation of H_2_S is toxic even though this gas is present in our body for a wide variety of functions. STS was not itself toxic to any cell types up to 500 μM under our experimental conditions. Furthermore, STS and NaSH did not affect the viability of any glial cell type (Additional file 1: Figure S1–S3). Therefore, both agents appear to be potentially suitable as pharmaceutical drugs in conditions such as Alzheimer’s disease and Parkinson’s disease.

## Conclusions

In this report, we have shown NaSH and STS to be potent anti-inflammatory agents and STS is 20–40 % less active than NaSH in most assays, but there are no qualitative differences and this would merely translate into modestly higher doses to achieve the same effect. What is important is that STS is an approved agent while NaSH must go through many regulatory steps before it could be approved. For the present, emphasis should be on STS as a potential broad spectrum therapeutic agent.

## Additional file

Additional file 1: Figure S1. Effect of NaSH or STS on cell viability changes after treatment for 2 days with LPS/IFNγ-activated THP-1 cells (A), IFNγ-activated U373 cells (B), LPS/IFNγ-activated human microglia (C) and IFNγ-activated human astrocytes (D) as followed by MTT assays. (A) THP-1 cells and (B) U373 cells: 12 h preincubation with NaSH or STS and (C) microglia and (D) astrocytes: 12 h preincubation with NaSH or STS. Values are mean±SEM, *n* = 4. One-way ANOVA was carried out to test significance. Multiple comparisons were followed with post-hoc Bonferroni tests where necessary. Note that there was no viability change when each compound was exposed to the cells in the presence of LPS/IFNγ or IFNγ. **Figure S2**. Effect of treatment with NaSH and STS on SH-SY5Y cell viability changes induced by LPS/IFNγ-activated THP-1 cell CM (A) or IFNγ-activated U373 cell CM (B) as followed by MTT assays (Protocol 2). A: THP-1 cells and B: U373 cells. After THP-1 cells and U373 cells were stimulated for 2 days with LPS/IFNγ or IFNγ, respectively their supernatants were transferred to SH-SY5Y cells. Then NaSH or STS was added. MTT tests were performed after 3 days. Values are mean±SEM, *n* = 4. One-way ANOVA was carried out to test significance. Multiple comparisons were followed with post-hoc Bonferroni tests where necessary. Note that there are no viability changes when all the compounds were exposed to SH-SY5Y cells after LPS/IFNγ-activated THP-1 cell CM (A) or IFNγ-activated U373 cell CM (B) were transferred. **Figure S3**. Effects of treatment with NaSH and STS on microglial and astrocytic viability changes in the presence or absence of stimulants. (A) Microglia: no stimulation, (B) Astrocytes: no stimulation, (C) Microglia: Stimulation and (D) Astrocytes: Stimulation. (A,B) After microglia and astrocytes were treated with STS and NaSH for 3 days MTT assays were performed. (C,D) After microglia and astrocytes were treated with STS and NaSH for 12 h stimulants were added. After 2 day incubation MTT assays were performed. Values are mean±SEM, *n* = 4. Note that NaSH and STS did not change SH-SY5Y cell viability in the presence or absence of stimulants in any concentration we tested. (DOC 99 kb)

## Abbreviations

CM: conditioned medium
GSH: glutathione
IFNγ: interferon-γ
IL-6: interleukin-6
LDH: lactate dehydrogenase
LPS: lipopolysaccharide
STS: sodium thiosulfate
TNFα: tumor necrosis factor-α
