# Supplementary material for: Sodium thiosulfate attenuates glial-mediated neuroinflammation in degenerative neurological diseases
Source: J Neuroinflammation. 2016 Feb 8;13:32. doi: 10.1186/s12974-016-0488-8 (PMC4746933; doi:10.1186/s12974-016-0488-8)
Supplement: Additional file 1: Figure S1. — Effect of NaSH or STS on cell viability changes after treatment for 2 days with LPS/IFNγ-activated THP-1 cells (A), IFNγ-activated U373 cells (B), LPS/IFNγ-activated human microglia (C) and IFNγ-activated human astrocytes (D) as followed by MTT assays. (A) THP-1 cells and (B) U373 cells: 12 h preincubation with NaSH or STS and (C) microglia and (D) astrocytes: 12 h preincubation with NaSH or STS. Values are mean±SEM, n = 4. One-way ANOVA was carried out to test significance. Multiple comparisons were followed with post-hoc Bonferroni tests where necessary. Note that there was no viability change when each compound was exposed to the cells in the presence of LPS/IFNγ or IFNγ. Figure S2. Effect of treatment with NaSH and STS on SH-SY5Y cell viability changes induced by LPS/IFNγ-activated THP-1 cell CM (A) or IFNγ-activated U373 cell CM (B) as followed by MTT assays (Protocol 2). A: THP-1 cells and B: U373 cells. After THP-1 cells and U373 cells were stimulated for 2 days with LPS/IFNγ or IFNγ, respectively their supernatants were transferred to SH-SY5Y cells. Then NaSH or STS was added. MTT tests were performed after 3 days. Values are mean±SEM, n = 4. One-way ANOVA was carried out to test significance. Multiple comparisons were followed with post-hoc Bonferroni tests where necessary. Note that there are no viability changes when all the compounds were exposed to SH-SY5Y cells after LPS/IFNγ-activated THP-1 cell CM (A) or IFNγ-activated U373 cell CM (B) were transferred. Figure S3. Effects of treatment with NaSH and STS on microglial and astrocytic viability changes in the presence or absence of stimulants. (A) Microglia: no stimulation, (B) Astrocytes: no stimulation, (C) Microglia: Stimulation and (D) Astrocytes: Stimulation. (A,B) After microglia and astrocytes were treated with STS and NaSH for 3 days MTT assays were performed. (C,D) After microglia and astrocytes were treated with STS and NaSH for 12 h stimulants were added. After 2 day incubation MTT assays were [file 12974_2016_488_MOESM1_ESM.doc]

**Additional file 1: FIGURE S1.**

**
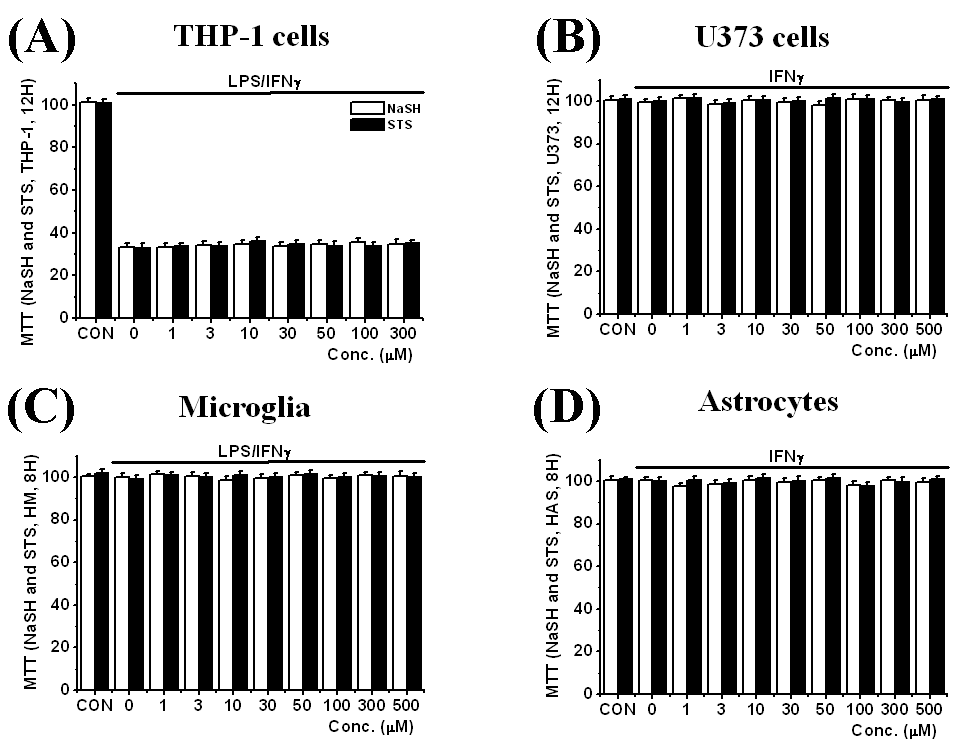
**

**FIGURE S1**. Effect of NaSH or STS on cell viability changes after treatment for 2 days with LPS/IFNγ-activated THP-1 cells **(A)**, IFNγ-activated U373 cells **(B)**, LPS/IFNγ-activated human microglia **(C)** and IFNγ-activated human astrocytes **(D)** as followed by MTT assays. **(A)** THP-1 cells and **(B)** U373 cells: 12 h preincubation with NaSH or STS and **(C)** microglia and **(D)** astrocytes: 12 h preincubation with NaSH or STS. Values are mean±SEM, n=4. One-way ANOVA was carried out to test significance. Multiple comparisons were followed with *post-hoc* Bonferroni tests where necessary. Note that there was no viability change when each compound was exposed to the cells in the presence of LPS/IFNγ or IFNγ.

**Additional file 1: FIGURE S2.**

**
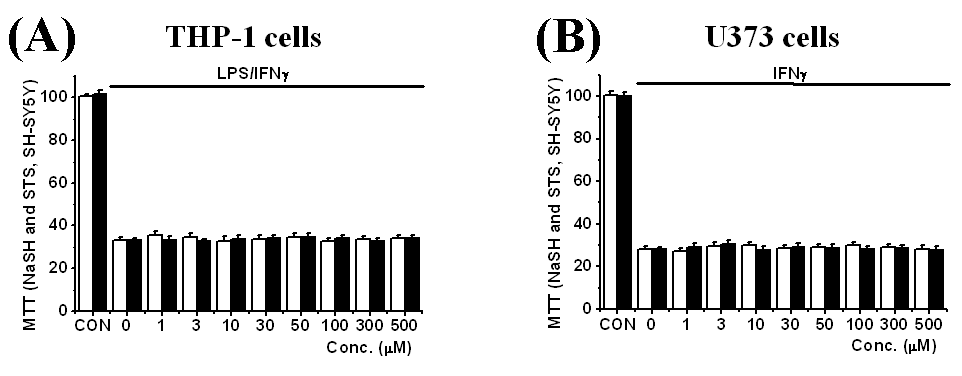
**

**FIGURE S2.** Effect of treatment with NaSH and STS on SH-SY5Y cell viability changes induced by LPS/IFNγ-activated THP-1 cell CM **(A)** or IFNγ-activated U373 cell CM **(B)** as followed by MTT assays (Protocol 2). **A**: THP-1 cells and **B**: U373 cells. After THP-1 cells and U373 cells were stimulated for 2 days with LPS/IFNγ or IFNγ, respectively their supernatants were transferred to SH-SY5Y cells. Then NaSH or STS was added. MTT tests were performed after 3 days. Values are mean±SEM, n=4. One-way ANOVA was carried out to test significance. Multiple comparisons were followed with *post-hoc* Bonferroni tests where necessary. Note that there are no viability changes when all the compounds were exposed to SH-SY5Y cells after LPS/IFNγ-activated THP-1 cell CM **(A)** or IFNγ-activated U373 cell CM **(B)** were transferred.

**Additional file 1: FIGURE S3.**

**
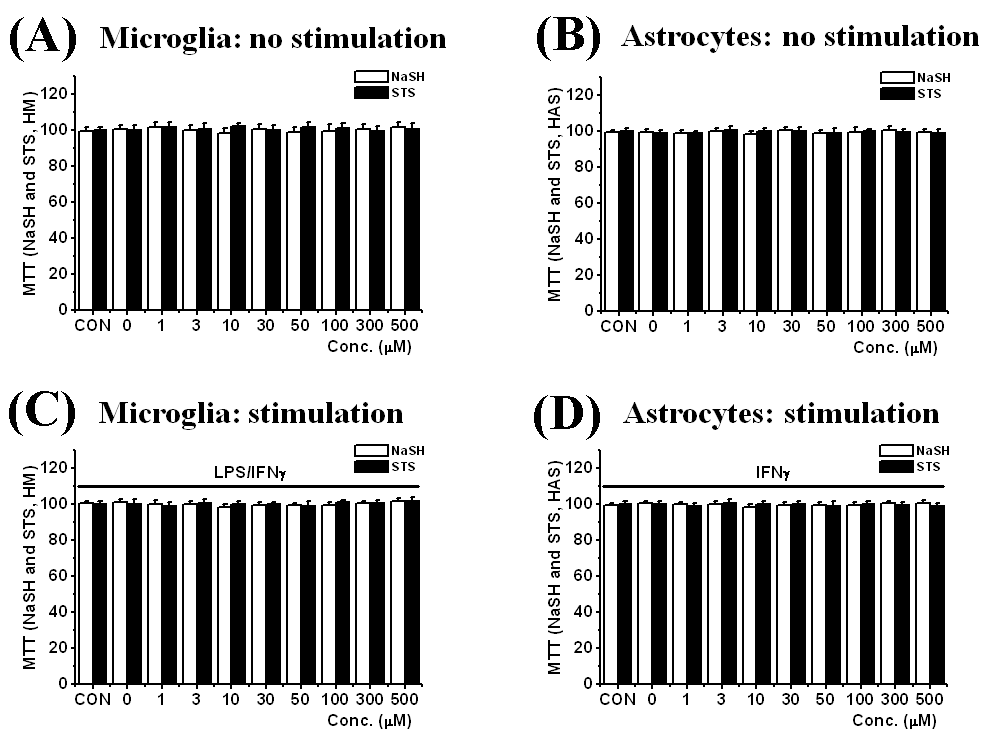
**

**FIGURE S3.** Effects of treatment with NaSH and STS on microglial and astrocytic viability changes in the presence or absence of stimulants. **(A)** Microglia: no stimulation, **(B)** Astrocytes: no stimulation, **(C)** Microglia: Stimulation and **(D)** Astrocytes: Stimulation. **(A,B)** After microglia and astrocytes were treated with STS and NaSH for 3 days MTT assays were performed. **(C,D)** After microglia and astrocytes were treated with STS and NaSH for 12 h stimulants were added. After 2 day incubation MTT assays were performed. Values are mean±SEM, n=4. Note that NaSH and STS did not change SH-SY5Y cell viability in the presence or absence of stimulants in any concentration we tested.
